# Genomic and transcriptomic sequencing of *Rosa hybrida* provides microsatellite markers for breeding, flower trait improvement and taxonomy studies

**DOI:** 10.1186/s12870-018-1322-5

**Published:** 2018-06-15

**Authors:** Weicong Qi, Xi Chen, Peihong Fang, Shaochuan Shi, Jingjing Li, Xintong Liu, Xiaoqian Cao, Na Zhao, Huiyuan Hao, Yajie Li, Yujie Han, Zhao Zhang

**Affiliations:** 1 Salt-Soil Agricultural Center, Institute of Agricultural Resources and Environment, Jiangsu Academy of Agricultural Sciences, Nanjing, 210014 China; 2 Institute of Plant Protection, Jiangsu Academy of Agricultural Sciences, Nanjing, 210014 China; 3 Beijing Key Laboratory of Development and Quality Control of Ornamental Crops, Department of Ornamental Horticulture, China Agricultural University, Yuanmingyuan Xilu 2, Beijing, 100193 China; 4 Nextomics Biosciences Co., Ltd., Wuhan, 430073 China

**Keywords:** Rose, Genome, Transcriptome, Simple sequence repeat, Transcription factor

## Abstract

**Background:**

*Rosa hybrida* is a valuable ornamental, food and medicinal crop worldwide, but with relatively limited molecular marker resources, especially for flower-specific markers. In this study, we performed genomic and floral transcriptomic sequencing of modern rose. We obtained comprehensive nucleotide information, from which numerous potential simple sequence repeat (SSR) markers were identified but were found to have high rates of amplification failure and PCR product redundancy.

**Results:**

We applied a filtering strategy for BLAST analysis with the assembled genomic sequence and identified 124,591 genomic and 2,292 EST markers with unique annealing sites. These markers had much greater reliability than those obtained before filtering. Additional BLAST analysis against the transcriptomic sequences uncovered 5225 genomic SSRs associated with 4100 transcripts, 2138 of which were associated with functional genes that were annotated against the non-redundant database. More than 90% of these newly developed molecular markers were polymorphic, based on PCR using a subset of SSRs to analyze tetraploid modern rose accessions, diploid *Rosa* species and one strawberry accession. The relationships among *Rosa* species determined by cluster analysis (based on these results) were in agreement with modern rose breeding history, whereas strawberry was isolated in a separate cluster, as expected.

**Conclusions:**

Our results provide valuable molecular-genetic tools for rose flower trait improvement, breeding and taxonomy. Importantly, we describe a reproducible organ-specific strategy for molecular marker development and selection in plants, which can be applied to other crops.

**Electronic supplementary material:**

The online version of this article (10.1186/s12870-018-1322-5) contains supplementary material, which is available to authorized users.

## Background

Rose belongs to the genus *Rosa* and the family *Rosaceae* [1]. The genus *Rosa* comprises ~ 200 species with a basic chromosome number (x) of 7 [2, 3]. More than half of the wild rose species worldwide are polyploid, ranging from 4n = 4× = 28 to 10n = 10× = 70 [4]. Rose is one of the most popular ornamental plants worldwide and has been cultivated as a garden plant for millennia. Roses have also been used as cut flowers and in perfume for hundreds of years, making rose an economically valuable crop in many countries. In China, besides their ornamental use, roses are also used as food and traditional medicinal crops. Chinese rose has been cultivated for ~ 5000 years. In the fourteenth century, Chinese rose germplasms were introduced into Europe by missionaries or merchants. Extensive hybridization between Chinese, European and Middle-Eastern roses and other wild *Rosa* species has formed the genetic basis of the “modern rose cultivars” [5]. As many as 30,000 to 35,000 of these cultivars, which are referred to as *Rosa hybrida* L., are currently being cultivated. Most of these cultivars are tetraploid (4×) [4]. A massive introgression of *R. chinensis* alleles remains in the *R. hybrida* genomes [6]. Recurrent flowering, flowering time, flower organ morphogenesis, flower color, flower scent and resistance to biotic and abiotic stress are important economic traits in modern rose breeding [4]. As with other crops, molecular markers are important tools for genetic research and breeding in rose with many uses, such as creating linkage maps, gene cloning and molecular marker-assisted breeding associated with these desired traits [7].

In 2003, Esselink et al. first identified simple-sequence-repeat (SSR) markers from enriched small-insert *R. hybrida* genomic libraries, from which 24 polymorphic sequenced tagged microsatellite site markers were isolated [8]. In 2004, Esselink et al. used some of these markers to identify allelic copies based on band intensity [9]. In 2006, Zhang et al. identified 30 polymorphic rose SSR markers, which were obtained from genomic DNA library construction followed by Southern blotting with a probe containing several repeat motifs [10]. In 2008, Oyant et al. identified 44 EST-SSRs and 20 genomic-SSRs from a rose bud-expressed sequence tag database comprising 2556 unigene sequences and a rose genomic BAC library with 384 clones [11]. In 2015, Koning-Boucoiran et al. created an Axiom SNP array containing approximately 68,000 SNP markers developed from a large collection of rose ESTs [12]. This microarray chip was later used to identify genes involved in floral color in rose [13] and tetraploid rose genome evolution [14]. Nonetheless, flower-specific molecular marker resources are not available for rose yet.

Here, we present a novel organ-specific strategy to develop SSR markers that are informative and codominant, as well as being low cost and reproducible on a large scale. Using next-generation sequencing technology, we sequenced genomic DNA and identified SSR markers from *R. hybrida* cultivar ‘Samantha’ (tetraploid); additional BLAST analysis against the flower transcriptomic sequences uncovered SSR markers associated with functional genes expressed in flowers. Compared to previous data, this new strategy generated an enormous number of SSR marker resources specific for flower organs of rose. Importantly, this strategy represents a novel method that has the potential to be applied to other plant species and provide organ-specific molecular markers. Identification of such organ-specific markers for the most valuable part of crops will lay a solid foundation for future studies about important organs traits.

## Methods

### Plant materials and isolation of DNA and RNA for sequencing

Genome DNA and floral transcriptomic RNA were isolated from Hybrid Tea Rose ‘Samantha’ leaves for paired-end sequencing on the Illumina 150 × 2 platform. ‘Samantha’ produces scented, medium-sized red flowers and dark green, leathery foliage, for which it is cultured worldwide [15]. Genomic DNA was isolated from young leaves of rose plants as described by Aldrich and Cullis (1993) [16], but with 1% (*w*/*v*) polyvinylpyrrolidone-10 added to the DNA extraction buffer. Total RNA was extracted from rose floral organs using a TRIzol RNA Purification kit (TaKaRa, China) following the manufacturer’s instructions. The RNA was treated with RNase-free DNase (Tiangen, China) to remove residual genomic DNA. First-strand cDNA synthesis was conducted using 20-μL reaction mixtures containing 1 μg of total RNA and oligo(dT) primers (TaKaRa, China).

### Library construction and Illumina sequencing

Illumina sequencing was conducted at NextOmics Ltd., in Wuhan, China. Genomic DNA was also fragmented into 100 to 400 bp segments for library construction. After validation on an Agilent Technologies 2100 Bio-analyzer, the library was sequenced on the Illumina HiSeq™ 2000 system (Illumina Inc., San Diego, CA) using the following workflow: template hybridization, isothermal amplification, linearization, blocking, sequencing primer hybridization and sequencing on the sequencer for read 1. After completing the first read, the template scan was regenerated in situ to enable a second read from the opposite end of the fragments. Once the original templates were cleaved and removed, the reverse strands were subjected to sequencing-by-synthesis, and mRNA with a poly(A) tail was isolated from 20 μg total RNA using Sera-mag magnetic oligo(dT) beads (Illumina). To avoid priming bias, the purified mRNA was first fragmented into small pieces (100–400 bp) using divalent cations at 94 °C for 5 min. Using random hexamer primers (Illumina), double-stranded cDNA was synthesized using a SuperScript Double-stranded cDNA Synthesis kit (Invitrogen, CA). The synthesized cDNA was subjected to end-repair and phosphorylation, and the repaired cDNA fragments were 3′ adenylated using Klenow Exo- (3′ to 5′ exo-, Illumina). Illumina paired-end adapters were ligated to the ends of these 3′-adenylated cDNA fragments. To select the proper templates for downstream enrichment, the products of the ligation reaction were purified on a 2% agarose gel. The cDNA fragments (~ 200 bp long) were excised from the gel. Fifteen rounds of PCR amplification were conducted to enrich the purified cDNA template using PCR primers PE 1.0 and 2.0 (Illumina) and Phusion DNA polymerase. The cDNA library was constructed using 200-bp insertion fragments.

### De novo assembly

Prior to assembly, a stringent filtering process of raw sequencing reads was conducted; the reads were stored in FASTQ format. Reads with > 10% of bases with a quality score of Q < 30 (Q30 quality control), non-coding RNA (such as rRNA, tRNA and miRNA), ambiguous sequences represented as “N”, empty reads and adaptor contamination were removed or filtered. Purified, high-quality transcriptome sequencing data were de novo assembled with SOAPdenovo2 using the De-Bruijn-Graph method and default settings, except for the k-mer value; the k-mer value with the best N50 size was selected for final assembly. The floral transcriptome was assembled using Trinity software.

### Functional annotation of transcripts

Selected transcript sequences were annotated by comparing them with sequences in public databases. Protein sequence similarities were determined via comparison against the NCBI (National Center for Biotechnology Information, http://www.ncbi.nlm.nih.gov/) non-redundant (NR) protein database.

### Development and detection of SSR markers

Simple sequence repeat (SSR) loci on the newly assembled genomic and transcriptomic contigs were detected using MISA (http://pgrc.ipk-gatersleben.de/misa/). The preference for identifying SSR loci was set to perfect motifs 2 bp to 6 bp in length, while the minimum number of motif repeats was 5. Primers were selected from the flanking sequences of the motif tandems with the Primer 3 program. The primers varied in length from 18 to 20 bp (optimal length: 20 bp), with GC contents ranging from 45 to 65% (optimal GC content: 50%). The preferred melting temperature was 60 °C, the minimum size of the PCR product was 120 bp and the maximum size was 400 bp.

### Evaluating the newly develop SSR primer pairs by in silico PCR

In silico PCR was conducted with the BLASTN program (ftp://ftp.ncbi.nlm.nih.gov/blast/executables/blast+/LATEST/). All of the newly developed primer pairs were annealed back to the assembled genome contigs. Any possible amplification was detected, and primer pairs producing multiple amplicons were filtered out. A second in silico PCR was carried out using the remaining primer pairs against the genome sequences of *Fragaria vesca* (woodland strawberry) [17], *Prunus persica* (peach) [18], *Malus × domestica* (apple) and *Rubus occidentalis* (raspberry) (https://www.rosaceae.org/). To identify primer pairs for potential functional SSR markers, the primers were annealed against the transcriptomic contigs. In silico PCR was then performed using genomic SSR primer pairs against the transcriptomic sequences using similar criteria, except that one base-pair mismatch at the 5′ end (none at the 3′ end) of the reverse primer was allowed.

### Evaluating the newly developed SSR markers by PCR

A total of 100 primer pairs were randomly selected from the newly developed genomic and EST SSRs and used in a laboratory PCR test with ‘Samantha’ genomic DNA as the template. ‘Samantha’ and 47 other accessions were used in a PCR test with 37 randomly chosen primer pairs. These 48 accessions included one diploid strawberry (*Fragaria vesca*), 28 tetraploid modern roses and 19 diploid old Chinese roses and other Rosa species. The genomic DNA was isolated as described above.

PCR amplification was conducted under the following conditions: DNA was denatured at 94 °C for 4 min, followed by 35–40 cycles of 94 °C for 30 s, Tm 55 °C–60 °C (specific for each primer, as shown in Additional file 1) for 30 s, 72 °C for 2 min and a final extension at 72 °C for 10 min. The PCR products were analyzed by electrophoresis on 8.0% non-denaturing polyacrylamide gels stained with ethidium bromide. The band sizes were determined against a DNA ladder.

### Data analysis

PIC values were calculated using the formula (PIC = 1-∑ (Pi)^2^), where Pi is the proportion of samples carrying the allele of a particular locus. Genetic distances were calculated using NTSYSpc (version 2.1 s) software with a coefficient of NEI72 (http://www.exetersoftware.com/cat/ntsyspc/ntsyspc.html). In addition, unweighted pair group method analysis (UPGMA) was performed for cluster analysis and to generate a representative tree plot with MEGA 7.0 software [19].

## Results

### Acquiring massive nucleotide information of *Rosa hybrida* by sequencing the ‘Samantha’ genome and floral transcriptome

**Fig. 1:**
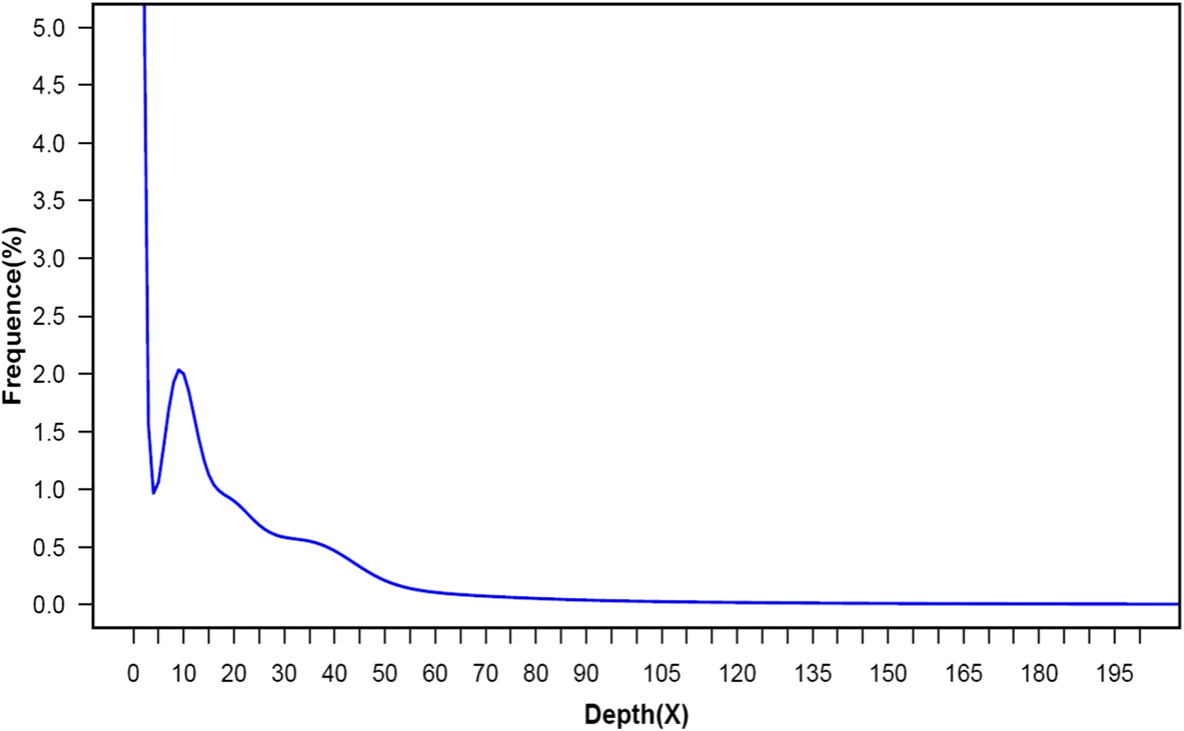
Kmer-17 depth distribution curve of the *Rosa × hybrida* genome sequence. A K-mer frequency distribution curve (k-mer = 17) using paired-ends with a 400 bp insert size is shown in the chart. The highest peak is located at a multiplicity of 9 (marked by the asterisk), and the estimated genome size based on the highest peak is 2.3 Gb

We began by sequencing the ‘Samantha’ genome (Table 1). A total of 212 million reads were generated from 32 Gb raw data. The GC content in the raw data was 40%, with a Q30 of 88%. After quality control, 160 million reads (23 Gb data) were retained, with a GC content of 39%. We estimated the genome size based on the frequency distribution curve of 17-mers obtained from Illumina short reads. A k-mer frequency distribution curve (k-mer = 17) constructed using paired-ends with a 400 bp insert size is shown in Fig.1. The highest peak was at a multiplicity of 9, and the estimated genome size based on the highest peak was 2.3 Gb. Based on this estimated genome size, the deduced coverage of the present genome sequence is approximately 37 × .

**Table 1:** Summary of de novo assembly of the genome and transcriptome data

	Genome		Transcriptome
	Contig	Scaffold	
Raw data	32.0G		7.2G
Number	2,445,344	554,534	71,532
N50	171 bp	1681 bp	1526 bp
Maximum Length	9114 bp	67,855 bp	12,858 bp
Average Length	180.2 bp	1311.3 bp	992 bp
Total Length	0.44 Gb	0.73 Gb	0.71 Gb

A total of 554,534 scaffolds of the genome were generated (with a total length of 0.73 Gb), with the longest scaffold 67.8 kb and an N50 length of 857 bp (Table 1). The total gap length in the scaffolds was 0.14 Gb, which were filled by Ns (n). There were also contigs (total length of 0.44 Gb) that did not form scaffolds. The N50 of these contigs was 171 bp, with a maximum length of 9.1 kb. The total length of the scaffolds and the isolated contigs was 1.17 Gb, which was similar to the predicted semi-genome size.

The ‘Samantha’ floral transcriptome sequencing results are also provided in Table 1. We obtained 47 million raw reads with a total length of 7.2 G. After Q30 quality control, 95.5% of the data remained, and the GC content was 46.7%. The remaining reads were de novo assembled with Trinity, which generated 71,532 contigs/transcripts with a total length of 71 Mb. In 2015, Koning-Boucoiran et al. performed a de novo assembly based on a tetraploid rose floral transcriptome (sequenced using Illumina) and generated 78,000 transcripts [1], which is comparable to the present results. The N50 length of these transcripts was 1.5 kb, and the average length was 992 bp, with a maximum transcript length of 12.8 kb and a deduced coverage of ~ 100 × .

### High throughput simple sequence repeat molecular marker development for *R. hybrida*

A total of 250,690 SSR loci were identified from the genome sequence, including 151,047 from scaffold sequences and 99,643 from contigs. On average, one SSR locus was detected for every 4.8 kb of genomic sequence. Meanwhile, 22,352 SSR loci were called from the 126,220 transcript sequences (97 Mb), with one SSR locus in each 4.3 kb of transcriptomic sequence. Thus, the frequency of SSRs in the genome and transcriptome were comparable.

**Fig. 2:**
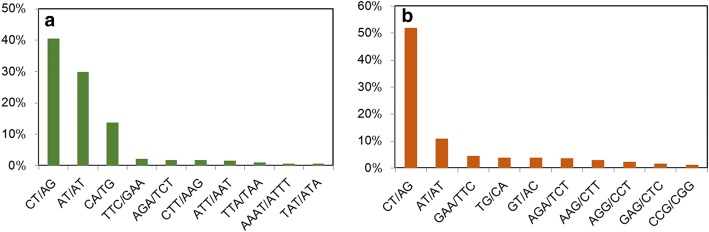
Motif distribution of the genomic and EST SSRs. Distribution of the top 10 motifs in genomic SSRs (**a**) and EST-SSRs (**b**). The most common motif in genomic and EST- SSR is the dinucleotide motif AG/CT. The X-axis represents the motif sequence, and the Y-axis represents the number of detected SSRs

A total of 807 different SSR motifs were detected from the genomic sequence and 144 from the transcriptome (Additional file 2). The top-10 most abundant SSR motifs are shown in Fig. 2, all of which are either di-nucleotide or tri-nucleotide. Among genomic SSRs, the top-3 motifs are CT/AG (40.5%), TA/TA (29.8%) and CA/TG (13.6%), while among transcriptome SSRs, CT/AG (51.9%) is the most abundant, followed by TA/TA (10.9%) and TTC/GAA (4.5%). The most abundant tri-nucleotide motif is TTC/GAA, which ranked fourth in the genome.

Among these newly identified rose SSR loci, 65.3% of the genomic SSRs and 72.1% of the transcriptomic SSRs meet the criteria of primer designation. In total, 146,919 genomic and 9018 transcriptomic primer pairs were obtained, i.e., almost 15-fold more genomic SSR vs. transcriptomic markers. The genomic and EST primers sequences are listed in Additional files 3 and 4, respectively.

A preliminary test of 100 randomly selected primers showed that 1) among the genomic primers, 54 produced multiple bands, 45 produced a single, clear amplicon and one yielded no identifiable band on the gel; 2) the results were worse for EST SSRs, as 79 failed to produce amplicons or generated ambiguous PCR products on the gel and the remaining SSRs produced multiple bands. These results suggest that the newly developed EST SSR markers are of lower quality than those from the genome. It is common that EST SSRs have lower chance than genomic SSRs to produce clear amplicon.

### Eliminating the SSR primer-pairs with residue amplicon facilitated by in silico PCR

In silico PCR against de novo assembled sequences showed that among the 146,919 genomic SSR primer pairs, 124,591 uniquely bound to the sites from which they were called, with the remaining ones carrying the risk of unexpected amplification. A similar test indicated that 2292 of the 9018 EST-SSR primer pairs amplified unique fragments against the genome sequences. In summary, among the newly designed markers, 84.8% of genomic SSR primer pairs and 25.4% EST SSR primer pairs produced a single amplicon; these primer pairs were retained and remaining ones were filtered out. The sequences of these primers are listed in Additional file 5.

We arbitrarily selected 100 each of the remaining genomic and transcriptomic primer pairs and performed PCR using ‘Samantha’ genomic DNA, followed by gel electrophoresis. We observed clear bands for 90 and 93 genomic and EST primer pairs, with 75 and 66 producing single bands, respectively. Therefore, selecting markers with unique annealing sites in the genome sequences yielded markers with greatly improved reliability.

### Potential transformability test in the newly developed genomic and EST SSRs in other *Rosaceae* species (*Fragaria vesca*, *Prunus persica*, *Malus domestica* and *Rubus occidentalis*)

**Fig. 3:**
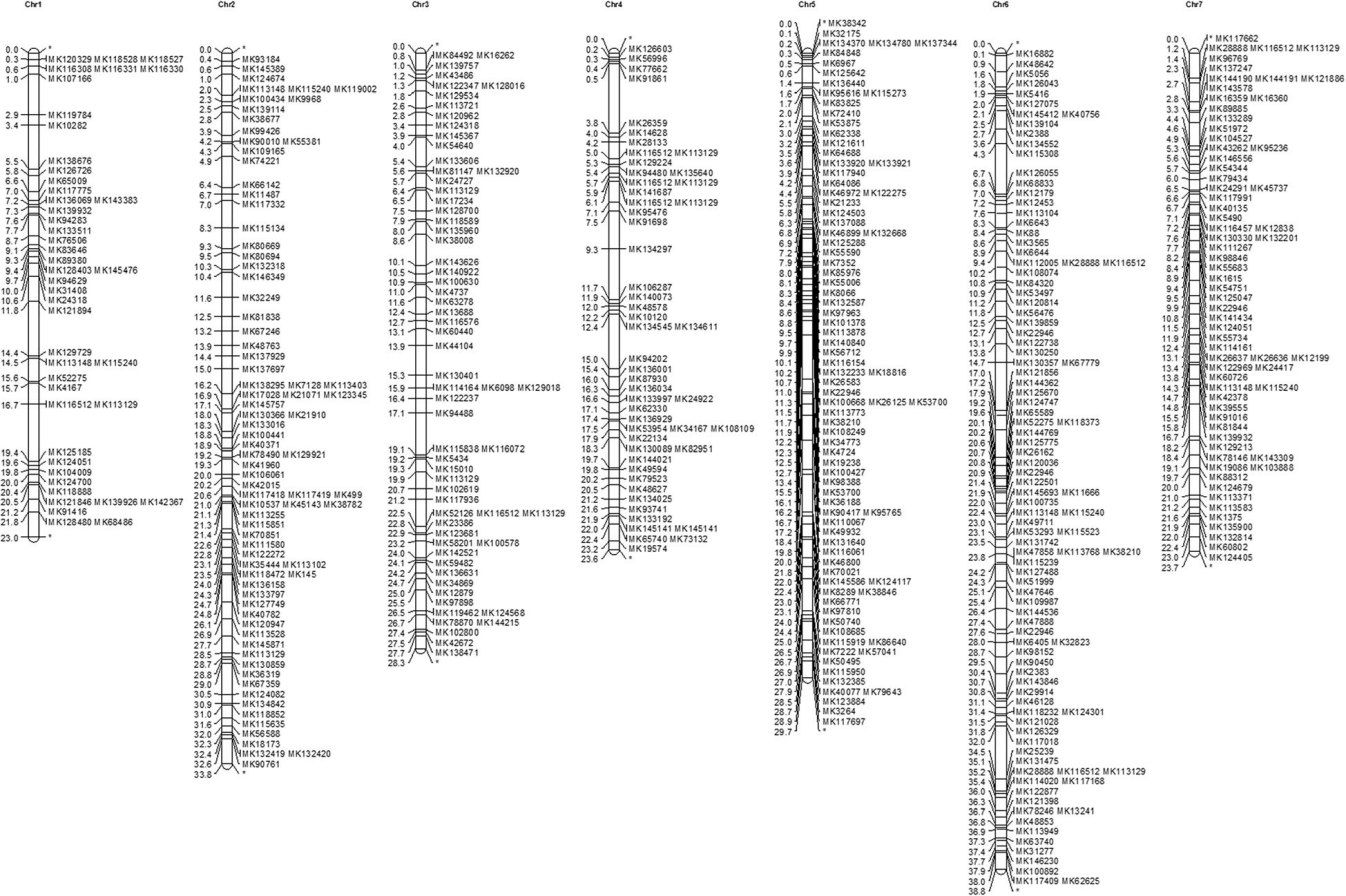
Distribution of rose genomic SSRs in the strawberry genome. Distribution of rose genomic SSRs in the genome of *Fragaria vesca*. The *F. vesca* genome contains seven chromosomes, in which a total of 475 rose genomic SSR markers were mapped onto 495 sites

Rose, strawberry and raspberry belong to the subfamily *Rosoideae*, while apple and peach belong to the subfamily *Spiraeoideae*. As shown in Tables 2, 462 genomic primer pairs and 156 EST primer pairs could be mapped onto the strawberry (*Fragaria vesca*) genome; 360 genomic and 112 EST primer pairs onto the raspberry genome; 27 genomic and 6 EST primer pairs onto the apple genome; and 27 genomic and 6 EST primer pairs onto the peach genome. The distribution of these newly developed genomic SSR markers on the *F. vesca* genome is shown in Fig. 3. A total of 495 annealing sites were detected on the seven strawberry chromosomes. The strawberry genome is approximately 200 Mb, and the average distance between two neighboring markers is 0.4 Mb. The primers that were mapped onto the peach, apple, raspberry and strawberry genomes, as well as their locations, are listed in Additional file 6.

**Table 2:** Validation of the genomic and EST-SSRs developed de novo from rose in other Rosaceae species (*Fragaria vesca*, *Prunus persica*, *Malus × domestica* and *Rubus occidentalis*)

	Genomic SSR	EST-SSR
Total	124,591	2292
Strawberry	462	156
Raspberry	360	112
Apple	28	7
Peach	27	6

### Identifying potential genes targeted markers associated with rose floral organ development from the newly developed massive SSR primer-pair set

As described above, we identified 2292 EST SSRs from 2292 independent EST contigs. We performed BLAST analysis of these 2292 contigs and annotated them against the non-redundant NCBI library. Only 33 contigs matched functional genes in the library, and thus, the corresponding SSR primer pairs were considered to be gene-targeted markers (GTMs) [20] (Additional file 7). To identify more GTMs, we tested the 124,591 validated genomic SSR primer pairs by in silico PCR against the de novo assembled floral transcriptome contigs, finding that 5225 of the primer pairs annealed to 4100 EST contigs (Additional file 7). Further BLAST analysis (as described above) revealed that 2748 of these contigs matched 2138 independent functional genes in the NR library. A total of 3212 genomic SSR primer pairs annealed to these 2748 EST contigs. Thus, 3245 SSR primer pairs were identified as GTMs, according to the floral transcriptome. The sequences of these primer pairs are listed in Additional file 7, as well as the relevant genes and their functions. The SSR primer-pair dataset generated in the present study likely includes additional GTMs, which should be identified using other transcriptome sequences. Thus, using this strategy, we identified SSR primer pairs specifically associated with the rose floral organ, which is the most valuable part of this ornamental crop.

### Taxonomic analysis of a rose germplasm collection using the newly developed SSR markers is in agreement with breeding history of modern rose

Among 37 randomly selected PCR pairs (primer information is listed in Additional file 1), 18 were associated with different floral transcripts, including eight that could be functionally annotated (Additional file 1). We combined the selected markers with independent DNA samples from 48 rose germplasms, including 30 tetraploid rose accessions (29 *R*. *hybrida* accessions and a *R*. *virginiana*) and 17 diploid accessions. The diploid accessions included four *Rosa chinensis*, four *Rosa odorata*, two *Rosa rugosa* and two *Rosa laevigata* accessions. Other *Rosa* species, including *R. rubus*, *R. roxburghii*, *R. cymosa*, *R. gigantea* and *R. banksiae*, were also included, as was woodland strawberry (*F. vesca*), which was also used to test the newly developed markers, as shown in Fig. 3. Information about these accessions is provided in Table 3. A total of 106 alleles were detected in the accessions using the selected markers. Thirty-three markers showed polymorphism, with PIC values ranging from 0.04 to 0.86, while the four remaining markers also showed clear banding patterns on the gel but no polymorphisms. There was no difference in PIC value between markers associated with the transcriptome and those that were not.

**Table 3:** Materials used for genetic diversity testing

#	Name	Ploidy	#	Name	Ploidy
1	Variegata Di Bologna	4×	25	Angela	4×
2	First Blush	4×	26	*R. rugosa* var. *rosea*	2×
3	Showtime	4×	27	Baby Romantica	4×
4	Xuelian	4×	28	Samantha	4×
5	Star Profusion	4×	29	*R. rubus*	2×
6	Cynthia	4×	30	*R. odorata* var. erubescens	2×
7	Rose Gaujard	4×	31	*R. chinensis* alba	2×
8	Rose de Rescht	4×	32	*R. roxburghii*	2×
9	Green Ice	4×	33	*R. chinensis* ‘Tu Wei’	2×
10	Hudiequan	4×	34	*R. cymosa*	2×
11	Cherry	4×	35	*R. gigantea*	2×
12	*R. rugosa* var. *alba*	2×	36	*R. banksiae*	2×
13	Sweet Pretty	4×	37	*R. laevigata* ‘Red Flower’	2×
14	Geraldine	4×	38	*R. virginiana*	4×
15	Golden Edge	4×	39	*R. odorata* var. *gigantea*	2×
16	Qingge	4×	40	*R. laevigata*	2×
17	Dainty Bess	4×	41	*R. odorata* var. spontanea ‘Pink Blush’	2×
18	Beijinghong	4×	42	*R. chinensis* ‘Leaflet Climber’	2×
19	Xiang Yi	4×	43	*R. odorata* ‘Hume’s Blush Tea-scented China’	2×
20	Regensberg	4×	44	*R. chinensis* ‘Old Blush’	2×
21	Xiang Fei	4×	45	*Fragaria vesca*	2×
22	No. 26	4×	46	Camara	4×
23	Elle	4×	47	Red Naomi	4×
24	Tian Mi De Meng	4×	48	Black Magic	4×

Agarose electrophoresis of the PCR products also revealed that 34 of these primer pairs yielded bands amplified in ‘Samantha’. Among these, 27 markers produced mono-allelic patterns, while six were di-allelic. The marker Geno-MK113936, which is associated with a gene that might encode a receptor-like protein kinase, had the highest PIC value and was tetra-allelic. Although the three remaining primer pairs gave no amplicon in ‘Samantha’, they produced clear bands in other accessions.

**Fig. 4:**
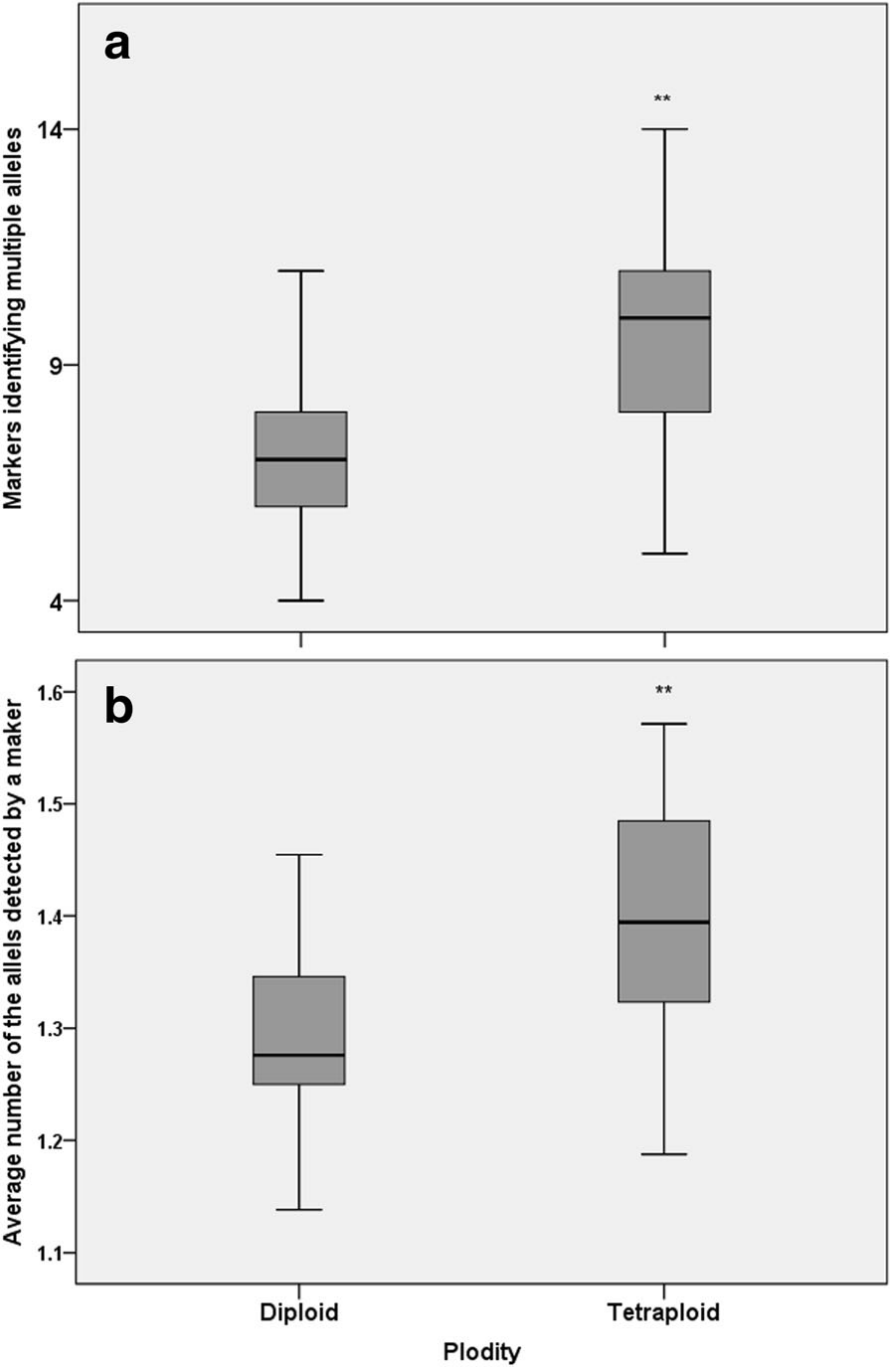
Alleles detected by markers in diploid and tetraploid accessions. More markers detected multiple alleles in tetraploid rose than in diploid rose (**a**). The average number of alleles detected by a marker was higher in tetraploid rose than in diploid rose (**b**). The asterisk indicates significant differences (one-way ANOVA: *p* < 0.05) between the diploid and tetraploid accession

Interestingly, more markers detected multiple alleles in tetraploid rose than in diploid rose (Fig. 4a); the average number of alleles detected by a marker in tetraploid rose was higher than in diploids (Fig. 4b), both of which were significant (one-way ANOVA: *p <* 0.05).

**Fig. 5:**
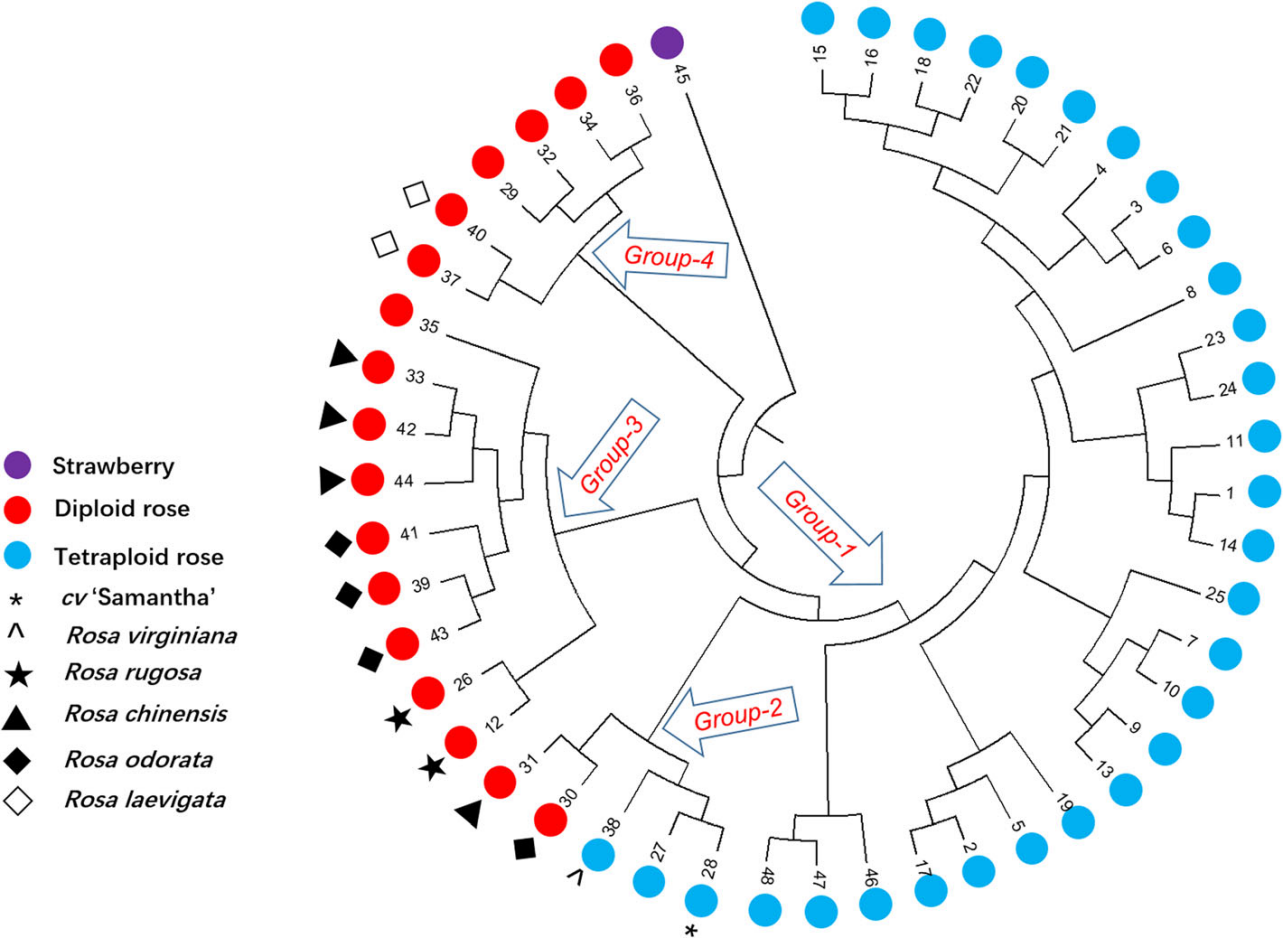
Tree plot of genetic diversity based on PCR results using 37 SSR markers. Tree plot derived from UPGMA cluster analysis using the NEI72 coefficients of SSR markers. The UPGMA (unweighted pair group method analysis) tree plot was generated using Mega 7.0 cluster analysis based on the genetic distances (calculated using NEI72 coefficients with NTSYS software) of 48 independent accessions. Blue circles = tetraploid rose accessions; red circles = diploid accession; purple circles = strawberry accession. The accession codes are marked, and the names can be found in Table 3. * = cultivar ‘Samantha’

Based on these PCR results, we calculated the genetic distance between different germplasms based on NEI72 coefficients, which ranged from 0.07 to 1.37, with strawberry showing the greatest genetic distance from roses. The genetic distance data are shown in Additional file 8. We used unweighted pair group method analysis (UPGMA) [34] to cluster the accessions, as shown in the tree plot in Fig. 5. The overall gene diversity (*H*_*T*_) [21] were also calculated based on the PCR result, in modern rose *(R. hybrida)* it was 0.46, and of the other *Rosa* species examined (excluding strawberry) was 0.47.

## Discussion

**Fig. 6:**
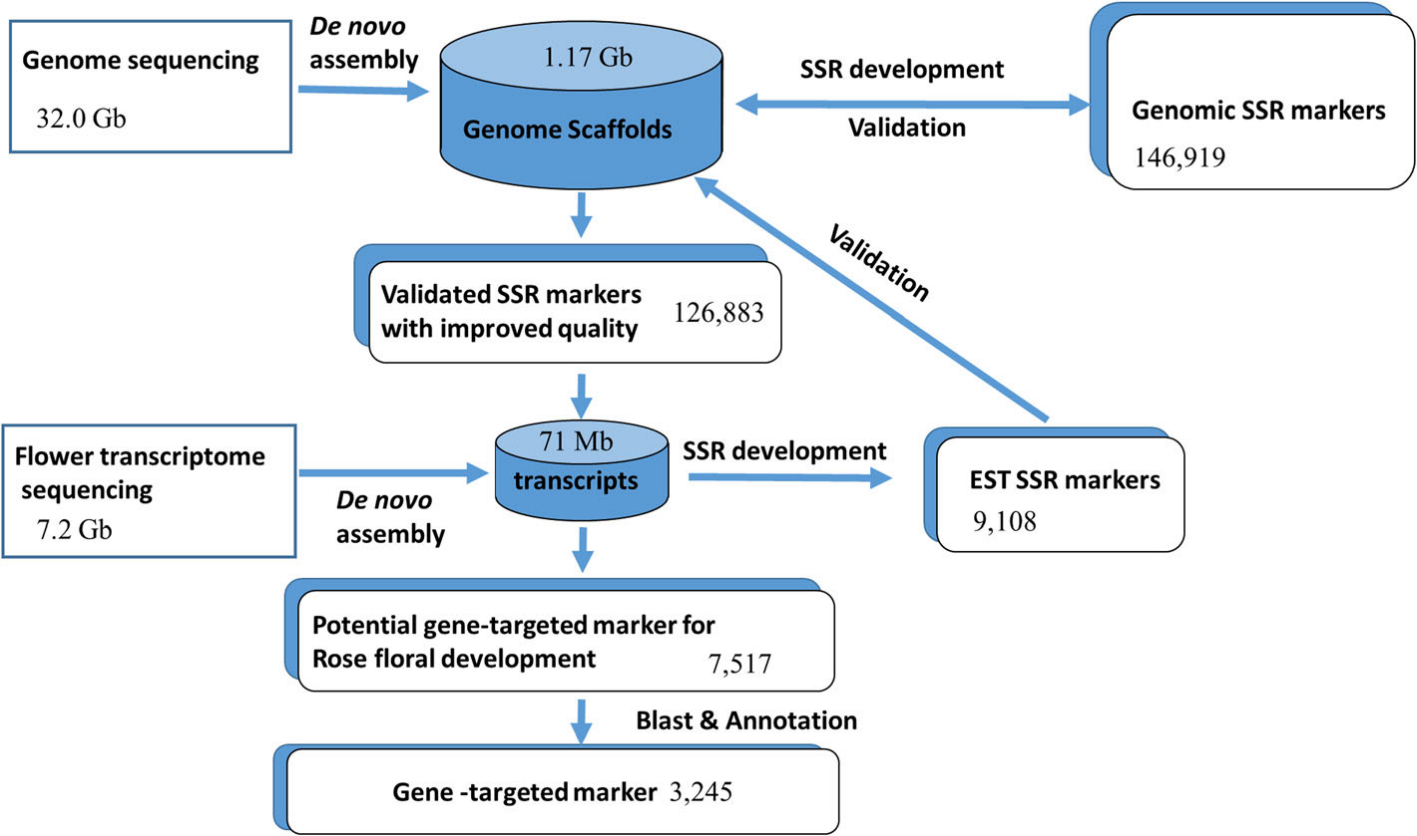
Workflow used in the present study. High-throughput sequencing was performed to generate 32 Gb and 7.2 Gb of raw reads for the rose genome and flower transcriptome, respectively. After de novo assembly, a total of 1.17 GB of genomic scaffolds, as well as 71 Mb of EST contigs were obtained. Altogether, 163,680 SSR markers were developed for these genome scaffolds and 16,345 for transcriptomic contigs. Further validation showed that 124,591 genomic and 2762 transcriptomic SSR markers (total of 127,353) had unique annealing sites on the scaffolds; these markers were of better quality than the primary markers. Based on BLAST analysis, 5225 genomic SSR markers were associated with the transcriptome. Together with the EST SSRs, 7987 SSRs were develop that could potentially be used as functional markers for rose floral development

In this study, we performed SSR marker mining in large scale based on new generation sequencing technology, and developed new strategy to identify organ-specific SSR markers using the rose genome and floral transcriptome. As shown in Fig. 6, we first generated SSR markers from both genomic DNA sequence and floral transcriptomic sequence. A filtering strategy involving in silico PCR testing against the assembled genome sequences filtered out 15.2% of the genomic SSR markers and most of the EST-SSR markers. Subsequently, we combined SSR primer pairs from both genome and flower transcriptome sequencing and eliminated the irrelevant ones (Fig. 6). This combined strategy allowed us to reduce the number of SSRs and to identify candidate SSR primer pairs responsible for the flower organ specifically. After BLAST analysis, among 126,882 newly developed SSR primer pairs, a total of 7987 primer pairs associated with the floral organ transcriptome were identified.

The result of modern rose genome sequencing indicated ‘Samantha’ having an estimated genome size as large as 2.3 Gb. By contrast, the estimated genome size of *R. hybrida* L. cv ‘Mme G. Delbard’ is 2.9 Gb based on 2C-value (2C = 2.94 pg) measured by flow cytometry (genome size (bp) = (0.978 × 10^9^) × 2C-value (pg)) [22]. The 2C-value of diploid Rosa species ranges from 0.94 pg to 1.14 pg [23], indicating that their genome size varies from 0.92 to 1.11 Gb. Since ‘Samantha’ is tetraploid, like ‘Mme G. Delbard’, the estimated genome size obtained in the present study is not unexpected. The tetraploidy of the genome and the read length limit of Illumina prevented us from obtaining a better map of *R. hybrida*. To improve this map, a technique allowing longer read lengths such as PacBio should be used, and additional genetic populations and genetic markers are needed. Information about these assembled scaffolds and contigs is freely available from the corresponding author upon request.

As showed by the results of SSR mining, the distribution of the abundance of different motifs is generally in agreement with that of other dicotyledonous plants [24–29]. However, GC/CG SSRs rarely occurred in the other dicots examined but comprise 0.2% of ‘Samantha’ rose genomic SSRs [30]. In comparison with other plants, modern rose has a relatively high number of SSRs compared to other plant species when mono-nucleotide motif tandems are not taken into account [30, 31]. The potential transferability of the newly developed SSR primer-pairs in modern rose, were tested by in silico PCR against genome sequences of *Fragaria vesca* (strawberry), *Prunus persica* (Pear), *Malus domestica* (apple) and *Rubus occidentalis* (raspberry). These results showed in Table 2, are in agreement with the phylogenetic relationships among *Rosaceae* genera reported by Potter et al., 2007 [32]: strawberry is closest to rose, followed by raspberry, while apple and pear are distantly related to rose. We also performed in silico PCR using these Rosa primer pairs against the genome of the model plant *Arabidopsis thaliana* [33]. Interestingly, no annealing site was detected on the chromosomes of this *Brassicaceae* plant species.

Later, we used some of the newly developed SSR primer pairs to evaluate the genetic diversity of rose and found them to be robust. Compared with the diploid accessions, more alleles were detected by the markers in the tetraploids. It implies that tetraploid roses generally show more multi-allelic patterns than diploids, which is in agreement with Esselink et al., 2014 [9]. Although the selected markers yielded amplification produced and showed polymorphism, this test was only preliminary. Further analysis is needed to investigate 1) the existence of null alleles; 2) the amplification of residue bands or stutter bands by gel electrophoresis and 3) the relationship between allele copy number and band intensity. The UPGMA analysis clearly shows that strawberry is isolated from *Rosa* species and that the modern roses are markedly separate from the diploid rose accessions. Compared with other tetraploid roses, ‘Samantha’ and ‘Baby Romantica’ share a closer relationship with diploid roses. Among the diploid roses, two *R. rugosa* accessions (#12 and #26) are adjoining in the tree plot, two *R. laevigata* are also close to each other, three (#33, #42 and #44) of the four *R. chinensis* accessions (#31, #33, #42 and #44) are clustered together and three (#39, #41 and #43) of the four *R. odorata* (#30, #39, #41 and #43) are clustered together. As shown in the phenetic tree, the *Rosa* species could be classed into four groups: Group 1 consists of modern roses; Group 2 contains two modern rose cultivars (‘Samantha’ and ‘Baby Romantica’) and *R. chinensis* var. ‘alba’, *R. odorata* var. erubescens and *R. virginiana*; Group 3 consists of *R. chinensis*, *R. odorata*, *R. rugosa* and *R. gigantea*; Group 4 contains *R. roxburghii*, *R. laevigata, R. cymosa, R. banksiae* and *R. rubus*. Four species in Group 2 and 3, *R. chinensis*, *R. odorata*, *R. rugosa* and *R. gigantea*, have contributed greatly to the development of modern roses [12]. These results are in agreement with earlier analyses as well as the breeding history of modern rose. The tree plot also indicates that tetraploid *Rosa* species *R. virginiana* is closely related to modern roses. Although little genetic analysis has been performed on *R. virginiana*, polygenetic analysis based on chloroplast *matK* sequences indicated that it shares a close relationship with *R. rugosa* [34]. In summary, the topological relationship indicated in the tree plot is in agreement with previous reports [4, 34, 35]. This result demonstrates that our newly developed markers using the filtering strategy are reliable for genetic analyses.

## Conclusions

The SSR primer-pair dataset developed in this study will serve as an excellent resource for researchers or breeders focusing on rose flowers, the most valuable part of this important crop. Importantly, our results demonstrate that this strategy using high-throughput sequencing technology allowed us to develop organ-specific SSR markers, which could be invaluable in other plant species as well.

## Additional file

Additional file 1: The SSR motifs detected in present research were showed, as well as the number and frequency of them. (XLSX 39 kb)

Additional file 2: The sequences of total genomic SSR primers were demonstrated in Additional file 2. (XLSX 34 kb)

Additional file 3: The sequences of total EST- SSR primers were demonstrated in Additional file 3. (XLSX 13308 kb)

Additional file 4: The sequences of SSR primers with single annealing site were demonstrated in Additional file 4. It was validated by in silico PCR. (XLSX 808 kb)

Additional file 5: Primers which can be mapped onto the peach, apple, raspberry and strawberry genomes are listed in Additional file 5. (XLSX 1286 kb)

Additional file 6: A total of 3245 genome SSR primer pairs and 33 of the EST were identified as gene-targeted makers, according to the floral transcriptome. The sequences of these primer pairs are listed in Additional file 6, as well as the relevant genes and their functions. (XLSX 46 kb)

Additional file 7: A total of 37 randomly selected PCR pairs that were engaged in laboratory PCR are listed in Additional file 7. (XLSX 199 kb)

Additional file 8: The genetic distance data base on the laboratory PCR are shown in Additional file 8. (XLSX 27 kb)

## Abbreviations

AFLP: Amplified fragment length polymorphism
EST: Expressed sequence tag
NR: Non-redundant protein database
PIC: Polymorphism Information Content
SNP: Single Nucleotide Polymorphism
SSR: Simple sequence repeat
UPGMA: Unweighted pair group method analysis
